# Assessing migraine patients with multifocal pupillographic objective perimetry

**DOI:** 10.1186/s12883-021-02239-z

**Published:** 2021-05-26

**Authors:** Eman N. Ali, Corinne F. Carle, Christian J. Lueck, Maria Kolic, Ted Maddess

**Affiliations:** 1 Eccles Institute of Neuroscience, the John Curtin School of Medical Research, Australian National University, Acton, ACT Australia; 2 Department of Neuroscience, King Fahad Specialist Hospital, Dammam, Saudi Arabia; 3 Australian National University Medical School, Acton, ACT Australia; 4 Department of Neurology, The Canberra Hospital, Canberra, ACT Australia

**Keywords:** Migraine, Trigeminovascular pathway, Melanopsin, Multifocal pupillography, Photosensitivity

## Abstract

**Background:**

To establish the effects of stimulating intrinsically-photosensitive retinal ganglion cells (ipRGCs) on migraine severity, and to determine if migraine produces objectively-measured visual field defects.

**Methods:**

A randomized, open labelled, crossover study tested migraineurs and normal controls using multifocal pupillographic objective perimetry (mfPOP) with 44 test-regions/eye. A slow blue protocol (BP) stimulated ipRGCs, and a fast yellow protocol (YP) stimulated luminance channels. Migraine diaries assessed migraine severity. Per-region responses were analyzed according to response amplitude and time-to-peak.

**Results:**

Thirty-eight migraineurs (42.0 ± 16.5 years, 23 females) and 24 normal controls (39.2 ± 15.2 years, 14 females) were tested. The proportion of subjects developing a migraine did not differ after either protocol, either during the 1st day (odds ratio 1.0; 95% confidence interval 0.2–4.4, *p* = 0.48) or during the first 3 days after testing (odds ratio 0.8; 95% confidence interval 0.3–2.1, *p* = 0.68). Migraine days/week did not increase following testing with either protocol in comparison to the baseline week (1.4 ± 1.6 pre-testing (mean ± SD), 1.3 ± 1.4 post-BP, and 1.3 ± 1.2 post-YP; *p* = 0.96), neither did other measures of severity. Migraine occurring up to 2 weeks before testing significantly lowered amplitudes, − 0.64 ± 0.14 dB (mean ± SE), while triptan use increased amplitudes by 0.45 ± 0.10 dB, both at *p* < 0.001.

**Conclusions:**

Stimulating ipRGCs did not affect migraine occurrence or severity. Pupillary response characteristics were influenced by the occurrence of a recent migraine attack and a history of triptan use.

## Background

Migraine is thought to be associated with cortical spreading depression (CSD) which consists of a propagated wave of profound depression in cerebral cortical neural activity preceded by transient neuronal activation. CSD is believed to underlie migraine aura and to be a trigger for the headache pain [1]. Light is a well-recognized trigger of migraine attacks [2], and photophobia is a core feature of the condition but the mechanism is not fully understood. Noseda et al. [3] have described a retino-thalamic pathway involving the intrinsically-photosensitive retinal gangion cells (ipRGCs), which may be responsible for photophobia [4, 5]. While the ipRGCs receive input from rods and cones they also contain the photosensitive pigment, melanopsin, and so are themselves sensitive to blue light in bright conditions [6]. They relay their responses to cells in the posterior thalamus, among other targets. The thalamic target cells also receive input from the trigeminovascular pathway, which is believed to carry the pain signals arising from the dura mater during migraine attacks [3], and send their output to the somatosensory cortex. Intense blue stimuli of up to 1 s are generally required to activate the melanopsin response of ipRGCs [6–8], and their slow responses govern the steady-state size of the pupil [9]. Human studies of the contribution of melanopsin to responses of the pupils have found stimuli of 1 to several seconds to be optimal [10–12]. A previous study conducted by Main et al. [13] observed that migraine patients found both short (blue) and long (red) wavelengths of light significantly more uncomfortable between attacks compared to normal controls and subjects with tension-type headache. However, those results were based on purely subjective measures and, overall, a method for objectively assessing ipRGC function in migraineurs would be useful.

Multifocal Pupillographic Objective Perimetry (mfPOP) is a developing diagnostic technique that assesses visual function objectively using pupillary responses. As is common in recent mfPOP studies, mfPOP protocols used here [14] assessed 44 locations in the visual fields of both eyes concurrently. By testing both eyes with 88 sets of independent stimuli, and recording the response of both pupils, the device can distinguish localized afferent and efferent defects [15, 16] within a few minutes. Alterations in pupillary responses have previously been described clinically in migraine patients: prolonged mydriasis has been reported during migraine attacks, sometimes persisting for up to 3 months after an attack, suggesting dysfunction of the parasympathetic supply of the pupils [17, 18]. However, conventional pupillometry has so far failed to confirm these findings [17]. Localized visual field defects have been reported in migraine using various forms of perimetry [19–22], but this is not a consistent finding [23]. Localized visual field changes in terms of both sensitivity and response delay have been reported using mfPOP in diabetic retinopathy [15], macular degeneration [24], glaucoma [14], multiple sclerosis [25], and concussion [26]. In simultaneous mfPOP and multifocal visual evoked potential (mfVEP) recordings higher-sensitivity is observed in extra-striate cortex in association with early-stage retinal disease [27], and attentional effects can also be quantified by mfPOP [28]. The ability to detect cortically-mediated effects suggested that mfPOP could be used to investigate migraineurs. Most mfPOP studies have used transiently-presented yellow stimuli designed to minimize the effects of lens brunescence and attention [28], although we have reported studies using red/green equiluminant mfPOP stimuli [29]. We have recently reported on blue mfPOP stimuli that strongly favor ipRGCs [14], which are 30 times longer in duration that traditionally transient mfPOP stimuli. Given the possibility that ipRGCs are involved in the pathogenesis of migraine [3] we decided to incorporate these long-duration blue stimuli in a study of migraine.

This study had three aims: first, to determine whether testing migraine patients with ipRGC-biased blue mfPOP stimuli would provoke/exacerbate symptoms when compared to the standard transient yellow stimuli designed to drive dynamic luminance-sensitive channels. Second, to determine the ability of either stimulus to detect pupillary abnormalities in the days and weeks following a migraine attack. Third, to detect any change of sensitivity or delay in the per-region responses to visual field stimulation in migraineurs compared to normal controls.

## Methods

### Study design and subjects

**Fig. 1:**
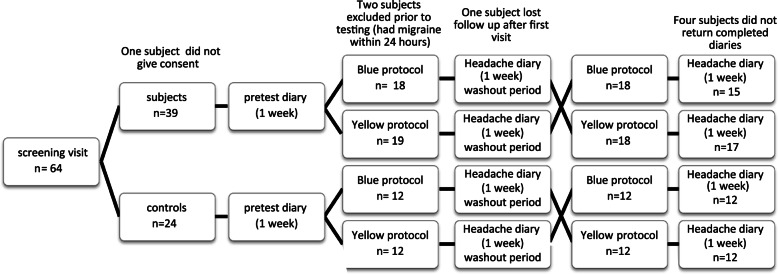
Study cross-over design. One-week headache diaries were completed before testing, before the 1-week washout period, and after the second test involving a crossover between yellow and blue stimuli

A randomized, controlled, open-label, crossover, single-site study was undertaken over 1 year (Fig. 1). Subjects with migraine were recruited from staff and students at The Australian National University and via local neurologists at The Canberra Hospital in Canberra, Australia. Informed, written consent was obtained from all subjects. The study conformed to the Declaration of Helsinki guidelines and was approved by both the Human Research Ethics Committee of the Australian National University (2012/278) and the ACT Health Human Research Ethics Committee (ETH.3.12.064).

Inclusion criteria comprised: 1) age above 18 years, 2) a clear diagnosis of migraine with or without aura according to International Headache Society criteria [30], 3) corrected visual acuity in both eyes better than 6/12. Exclusion criteria included: 1) a history of other visual or neurological disturbance that might affect visual assessment, 2) a history of epilepsy, 3) colour blindness, 4) pregnancy, or 5) medication that could affect pupillary responses including miotics, antidepressants and some common decongestants [31], 6) migraine headache occurring within the 24-h period before testing. The control group consisted of age- and sex-matched participants who underwent a standard eye examine by MK including slit-lamp and acuity, and had the same medical history exam as the migraineurs by EA.

Sample size calculation suggested that a total of 22 migraine subjects and 22 controls would be needed in order to detect an effect size of 40% increase in migraine headache or aura occurrence after testing with the mfPOP device. The power was set at 80% using a two-sided t-test at the level of *p* = 0.05. This calculation was done using the sample size formula for proportions [32].

A screening session to establish eligibility was performed for each participant during which background information regarding age at migraine onset, typical triggers, pattern, frequency and duration of migraine, presence of photophobia, other headaches, time since the most recent headache attack, and medication use (therapeutic or preventative) was obtained. Medications were divided according to class into: triptans, opioids, or over-the-counter (OTC) medications (NSAIDs, aspirin and paracetamol).

Subjects and controls were asked to complete a baseline headache diary for 1 week. Participants were then randomized using Research Randomizer software [33] to undergo one of the two mfPOP testing protocols: transient-yellow or slow-blue (Fig. 1). The randomized crossover design minimized the influence of confounding covariates because each patient acted as their own control. After each test a second diary was completed for a week followed by a washout period of a week. Subjects then underwent the other mfPOP protocol after which they completed a third headache diary. For ethical reasons, the use of subjects’ usual pain-relieving medications was permitted. This was felt to be likely to increase adherence to the study and also enabled evaluation of the effects of medication use before and after testing. All participants were advised not to smoke, drink caffeinated beverages, or consume alcohol for 6 h before mfPOP testing.

### Migraine diaries

A validated migraine headache diary devised by The Diagnostic Headache Diary Study Group [34] was used on the three occasions described above. Parameters recorded included whether the subject experienced a migraine headache (yes/no), severity on a scale of 1–3 (1 = not bad, 2 = quite bad, 3 = very bad), duration (estimated from the time the first symptoms were noticed until the time the headache finally subsided), characteristics (throbbing or compressing /unilateral or bilateral), associated factors (presence of aura, photophobia, phonophobia, nausea, vomiting), precipitating and relieving factors, as well as medication consumption (including type, dosage and frequency).

### mfPOP assessment

**Fig. 2:**
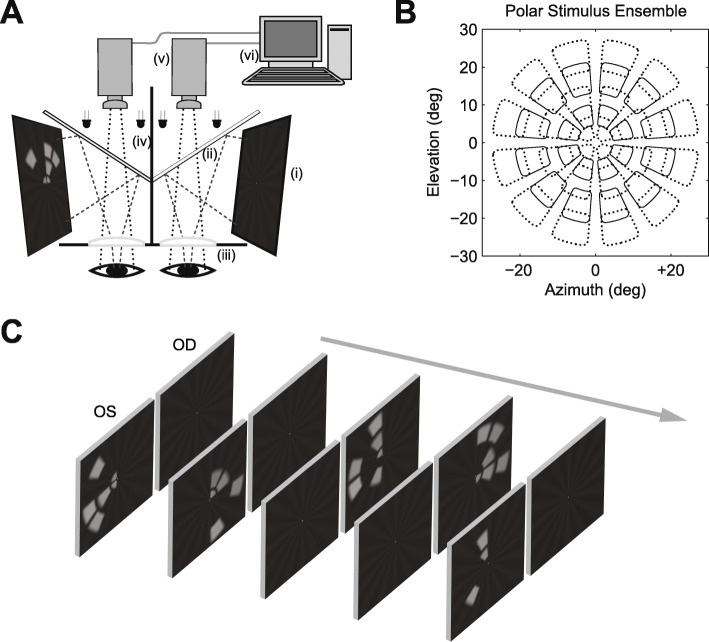
Objective measurement of the visual fields. **a** Schematic of the objectiveFIELD Analyser® (OFA). Stimuli are presented independently on two liquid-crystal displays (i). The images are reflected by two dichroic mirrors (ii) allowing infrared light to pass while reflecting shorter wavelengths. Plano-convex lenses set the viewing distance to optical infinity (iii). Each eye views only one monitor, the images being fused by the subject into a cyclopean view. Infrared illumination of the eyes is provided by infrared light-emitting diodes (iv) which allows monitoring of each pupil by infrared video cameras (v). Pupil diameters are extracted in real-time and recorded by a computer (vi). **b** The 44 stimulus regions/eye are arranged in a dartboard-like polar layout extending out to 30° from fixation. Stimuli are pseudo-randomly presented to each hemifield of each eye in a consecutive series. **c** shows the randomised volleys of independent dichoptic stimuli across a series of video frames of the test sequence. Along with a fixation cross a faint stationary background starburst pattern assists the subjects to fuse the images

All subjects underwent mfPOP assessment using the FDA-cleared objectiveFIELD Analyser® (OFA) prototype (Konan Medical USA, Irvine CA) (Fig. 2). Figure 2a shows a schematic of the device. Corrective lenses compensated for refractive errors. Trains of pseudo-randomly presented stimuli were presented at 44 possible locations/eye concurrently (Fig. 4b). The characteristics of the Yellow Protocol (YP) and the Blue Protocol (BP) are given in Table 1. The same stimuli have been used previously in a study of glaucoma and more details are provided there [14]. Aside from data presented there, the data of [35, 36] indicate that at the background and stimulus intensities used here pupil responses to blue stimuli are substantially driven by melanopsin. The 1 s duration of the BP stimuli is also optimal for such responses [10].

**Table 1:** Stimulus characteristics of the blue and yellow stimulus protocols

Stimulus protocol	CIE x,y colour coordinates	Maximum luminance (cd/m^2^)	Mean per-region interval (s)	Per-region duration (ms)	N repeats per region
**Blue (BP)**	0.145, 0.113	75	8	1000	45
**Yellow (YP)**	0.377, 0.464	150	4	33	90

**Fig. 3:**
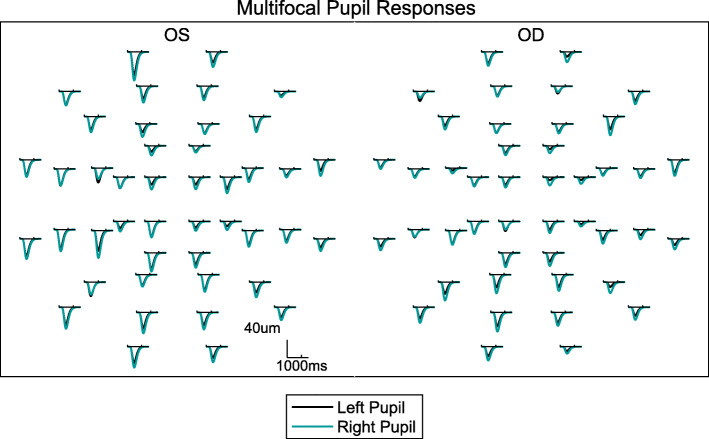
Example mfPOP response waveforms from an individual subject**.** The mean pupil responses to stimuli presented to each of the 44 test regions of the Yellow protocol were obtained from both eyes concurrently. Downward deflection indicates constriction, i.e. reduced diameter. The independent pseudo-random stimulation at each test-region means each waveform is a direct or consensual response that is independent of all the others. The black and teal traces represent the mean responses of the left and right pupils to 90 presentations at each location (Table 1)

The spatial layouts of YP and BP were identical: the locations where stimuli could occur comprised a dart-board like pattern consisting of 5 rings of stimuli extending to ±30°eccentricity of visual field (Fig. 2b). Figure 2c shows that over time the stimuli were presented in randomized *clustered volleys* [37]. The possible stimuli within a volley consisted of hemifield-sets of either the two rings defined by solid lines in Fig. 2b, or the 3 rings defined by dashed lines. Periodically, every 0.25 s/region for YP, and 0.5 s/region for BP, a given hemifield/ring set had the opportunity to be displayed, with 50% probability for any one stimulus location. This sequencing was designed to keep pupil sensitivity high and balanced over time [38]. When presented, individual stimuli remained on for different durations: 33 ms for YP, and 1000 ms for BP. Both protocols ran for 360 s, presented in 9 segments of 40 s duration to allow rest breaks. This meant that, on average, the mean interval between stimuli experienced by a given visual field region was 4 s for YP, and 8 s for BP. For YP and BP this meant each region was tested 90 or 45 times respectively. Examples of the resulting averaged pupil responses from a single YP test are presented in Fig. 3. The background illumination of 10 cd/m^2^ adapted rod photoreceptor responses. The display included a small central (binocular) red fixation cross. Trial lenses were provided but to provide tolerance to mis-refraction the stimuli contained no spatial frequencies above 2 cycles/degree.

### Data analysis

Analysis was conducted using MATLAB software (MathWorks, Natick, MA). Response waveforms – for both direct and consensual responses – from each region of the visual field were obtained and fitted to a log-normal function as follows:

$$ \mathrm{v}\left(\mathrm{t}\right)=\mathrm{A}\ \exp\ \left(-\kern0.5em {\left[\mathrm{In}\left(\mathrm{t}/{\mathrm{t}}_{\mathrm{p}}\right)\right]}^2/2{\sigma}^2\ \right) $$

where *v(t)* is the response waveform, A is the peak amplitude, *t* is the time at which each estimation is made, *t*_*p*_ is the time to peak, and σ is the width of the response [15, 29].

This allowed the characterization of the responses according to standardized amplitude (AmpStd) and time-to-peak. AmpStd represents any change in pupil size corrected to the mean diameter of the population rather than using absolute pupil size and was expressed in decibels (dB). It was derived from constriction amplitude as follows: *AmpStd = constriction amplitude (*μm*) × 3500/c*. Where *c* is the mean pupil diameter based on the value of a line fitted to the entire 360 s of pupil diameter data recorded during each test, and 3500 μm is the nominal population mean. AmpStd was used to overcome inter-subject variation in mean pupil diameter and also improved tolerance to non-circular pupils, ansocoria, age and some drugs [31]. The higher the AmpStd, the larger the magnitude of pupillary constriction.

Student’s t-test and Fisher’s exact test were used to compare baseline characteristics. Odds ratios, McNemar’s and Cochran’s q tests, and one-way between-subjects ANOVAs were conducted to compare the number of subjects developing migraine after each protocol – the primary outcome – and the association between mfPOP measures and other migraine parameters. Multivariate linear models were used to assess the independent effects of migraine parameters on the pupillary response.

The percentage area under the curve (AUC) of the receiver operating characteristic (ROC) plot was used as a measure of the power of mfPOP to predict migraine diagnosis i.e. it quantified the overall ability of mfPOP to discriminate between individuals with and without migraine. ROC plots were constructed for both AmpStd and time-to-peak in both protocols, using either the single worst region in each visual field (i.e. the one most deviating from normal) or the mean of the five worst regions, looking at either single eyes or at the asymmetry between anatomically-equivalent regions of the two eyes [15].

## Results

Forty migraine patients were screened and 39 enrolled. Thirty-eight subjects completed testing with both mfPOP protocols. Two subjects were excluded because they developed a migraine within the 24 h prior to testing, one patient withdrew after the first test, and four subjects did not return completed diaries. In all, 32 sets of completed migraine diaries were returned and analyzed. In addition, 24 age- and sex-matched controls were studied (Table 2). Median acuity was 6/6, one migraine subject was 6/12 OU. There was no significant difference in acuity between the control and migraine subjects.

**Table 2:** Subject characteristics

	Subjects (*n* = 38)	Controls (*n* = 24)
**Age (mean ± SD)**	42.0 ± 16.5	39.2 ± 15.2
**Male: Female**	1: 1.8	1: 1.5
**Migraine type after BP or YP**		–
**•With aura**	26 (72%)	
**•Without aura**	15 (42%)	
**Mean age of onset (years) (±SD)**	17.8 ± 9.11	–
**Mean disease duration (years) (±SD)**	24.3 ± 16.7	–
**Treatment**		–
**•Preventative**	8 (22%)	
**•During attacks**		
**Over the counter**	25 (69%)	
**Triptans**	14 (39%)	
**Opioids**	13 (36%)	
**Ergot**	2 (6%)	
**Mean attacks per month (±SD)**	2.62 ± 5.17	–
**Mean headache duration (hours) (±SD)**	11.75 ± 16.5	–
**Trigger**		–
**•Light**	15 (41%)	
**•Other**	32 (88%)	
**Photophobia**	34 (94%)	–

### Effects of mfPOP testing on migraine

Only one patient had difficulty completing the BP, reporting the occurrence of an aura at the end of the test. Otherwise, all patients reported no discomfort during testing apart from mild tearing due to insufficient blinking. The effects of testing on other migraine parameters are summarized in Table 3, which shows the same number of patients – four subjects (12.5%) – developing a migraine attack in the first day after testing with either BP or YP. The difference was not significant (odds ratio 1.0; 95% confidence interval 0.2–4.4, *p* = 0.48). The results remained non-significant for both protocols over the first 72 h, with 11 subjects (34.4%) developing migraine after the BP and 13 subjects (41%) after the YP (odds ratio 0.8; 95% confidence interval 0.3–2.1, *p* = 0.68). This period of 72 h was examined based on evidence that it may take up to 48 h following a trigger for a migraine to occur [39]. Although migraines occurred after testing the number of migraine days/week was not significantly increased relative to pre-testing: 1.4 ± 1.6 pre-testing (mean ± SD), 1.3 ± 1.4 post-BP, and 1.3 ± 1.2 post-YP (*p* = 0.96 for both). Other migraine parameters including attack severity, attack duration, and percentage of patients taking medication before and after each test were also not significantly different.

**Table 3:** Effects of mfPOP stimulation on migraine headache severity parameters

Parameter	Pre-testing	Post-BP	Post-YP	*P*-value
Patients experiencing migraine in the 1st day post testing, no. (%)	–	4 (12.5%)	4 (12.5%)	0.48^a^
Patients experiencing migraine within 3 days post testing, no. (%)	–	11 (34.4%)	13 (41%)	0.68^a^
Migraine days/week (mean ± SD)	1.4 ± 1.6	1.3 ± 1.4	1.3 ± 1.2	0.96^b^
% of patients experiencing ‘quite bad’ to ‘very bad’ migraine	34%	25%	43%	0.10^c^
Mean attack duration (hours)	1.41	1.09	1.07	0.71^b^
% of patients taking medication	50%	50%	53%	0.93^c^

^a^McNemar’s test

^b^One way ANOVA

^c^Cochran’s Q test

### Changes in mean mfPOP response characteristics

Results for the mean (across all regions, eyes and pupils) AmpStd, time-to-peak and pupil diameter are summarized in Table 4. The pupil diameters were not different between control subjects and migraineurs, but the diameters for yellow stimuli were significantly larger than for blue (t = 8.6, *p* < 0.001). Being the means across regions etc. the results for AmpStd and time-to-peak mimic what might be obtained from a single large Ganzfield stimulus as is used in some pupil studies. No significant differences were found between patients and controls for either BP or YP.

**Table 4:** Mean BP and YP response characteristics of controls and migraineurs

		Control (mean ± SD)	Migraine (mean ± SD)
**Blue protocol**	**AmpStd (dB)**	12.1 ± 2.55	11.8 ± 2.49
	**Time-to-peak (ms)**	594.9 ± 53.3	604.5 ± 46.8
	**Pupil Diameter (mm)**	2.68 ± 0.29	2.77 ± 0.36
**Yellow protocol**	**AmpStd (dB)**	12.3 ± 1.77	11.9 ± 1.90
	**Time-to-peak (ms)**	493.5 ± 25.0	495.2 ± 21.9
	**Pupil Diameter (mm)**	3.41 ± 0.51	3.51 ± 0.74

In the absence of clear effects on the whole visual field, we next used a linear model to investigate localised defects. To minimise the effects of multiple comparisons we sorted the 44 regions in order of deviation from normal performance and then took the means of the 22/44 worst-performing regions as inputs to the linear model. This provided an assessment that made no a priori assumptions about any particular arrangement of localised visual field defects.

We fitted a factor for each of 3 increasingly long periods in which migraine occurred before testing, gender, age, medications, and other headaches. Of the various drugs, only triptans showed a significant effect. Overall, the closer a migraine attack occurred prior to the time of testing, the more negative the effect upon AmpStd, i.e. there was reduced sensitivity (Table 5). The greatest reduction was seen if the attack occurred within the week prior to testing, followed by 2 weeks and, lastly, 2 months before testing. The use of triptans was associated with a significant increase in AmpStd in both YP and BP (0.45 ± 0 .09 dB, and 0.48 ± 0.07 dB, respectively, *p* < 0.001). We fitted the identical model for times-to-peak. In that model Triptan use was not significant, and other headaches and being female were less significant. The main effects of interest were that a migraine within the previous 2 weeks was associated with faster responses: BP 21.9 ± 3.50 ms, and YP 29.7 ± 1.77 ms (*p* < 0.001 for both). Both more recent and more distant migraines were associated with smaller changes in delay. There was no *predictive* association with a migraine that was about to occur in the week following testing, i.e. no changes in the pupillary response were seen before a migraine attack that was about to happen. There was no association with disease duration, age of onset, triggers or photosensitivity.

**Table 5:** Independent effects of headache on AmpStd in migraine subjects from a linear model

	Blue protocol		Yellow protocol	
	dB ± SE	*P* value	dB ± SE	*P* value
**(Reference)**^a^	(−1.04 ± 0.10)	–	(− 0.92 ± 0.08)	–
**Migraine within last 60 days (*n*** **= 28)**	−0.45 ± 0.16	0.004	−0.08 ± 0.12	0.994
**Migraine within last 2 weeks (*n*** **= 24)**	− 0.64 ± 0.14	< 0.001	−0.65 ± 0.14	< 0.001
**Migraine within last week (*n*** **= 19)**	− 0.80 ± 0.10	< 0.001	−0.57 ± 0.08	< 0.001
**Triptan use (*n*** **= 13)**	0.45 ± 0.10	0.006	0.49 ± 0.07	< 0.001
**Other headaches (*n*** **= 2)**	2.39 ± 0.25	< 0.001	−1.16 ± 0.14	< 0.001
**Female gender (*n*** **= 22)**	0.06 ± 0.10	1.000	0.41 ± 0.08	< 0.001
**Age (dB per decade)**^b^	−0.28 ± 0.03	< 0.001	− 0.14 ± 0.02	< 0.001

*P*-values are Bonferroni corrected

^a^Male patients aged 40 years without migraine in more than 60 days and no triptan use

^b^Age was in decades relative to the median age of 40 years

### Visual field defects detected by the mfPOP

For each eye we derived deviation from normal performance, akin to the total deviations of a standard perimeter. The deviations were converted to Z-scores. Especially for BP, the observed deviations from normal appeared to be fairly random, with no obvious clinically-relevant features such as homonymous defects. To look for any consistent patterns we computed the means of the Z-scores across pupils and eyes and then converted those to *p*-values at *p* = 0.05, 0.02, 0.01 and 0.005 as is common for perimeters. Before averaging the polar mfPOP results were converted to a 24–2 pattern in order to facilitate comparison with standard perimetry. Details of the procedure have been provided elsewhere [40]. Given the results of the linear model, the averages for patients with different times since their last migraine were computed.

**Fig. 4:**
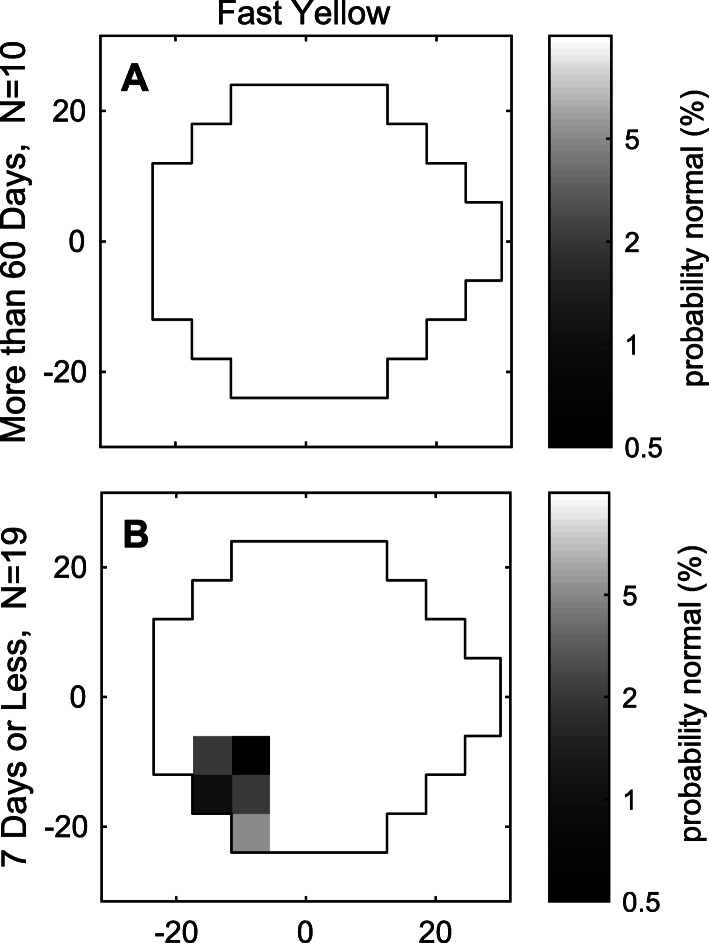
Average visual field defects for yellow protocol for **a** last migraine longer ago than 60 days before testing, and **b** last migraine within 7 days or less. Before averaging across data for eyes and pupils the z-scores were converted to a 24–2 format to make the results more comparable to standard perimetry. Data from right eyes were flipped to resemble left eyes so that corresponding parts of the fields could be averaged. Hence, while the data from 10 and 19 subjects were used for a and b, respectively, the actual number of fields averaged in each figure was 40 and 76 as this included fields for both eyes and both pupils. Despite 76 fields being averaged b shows a consistent inferior-temporal scotoma. The blue protocol data showed no consistent scotomas

For BP, no defect survived the averaging, i.e. there was no consistent defect in any part of the visual field data following a pre-test migraine. For YP, however, patients with a migraine 1 to 2 weeks before testing demonstrated consistent inferotemporal defects. Figure 4 shows the average YP results for patients with a migraine longer ago than 60 days (Fig. 4a) and for those with a migraine within the last week (Fig. 4b). For subjects with migraines within the last 14 days, the 4 most significant regions of Fig. 4b were at the 5% level.

### The power of mfPOP to predict the diagnosis of migraine

We next investigated the diagnostic power of BP and YP by examining the percentage area under the curve (%AUC) of the receiver operating characteristic (ROC) curve when comparing control eyes to patient eyes. To compare data based on the entire visual fields of every eye, we collapsed the direct and consensual responses of every region using weightings based on the signal-to-noise ratios of each region [15]. Table 6 shows the results comparing the 24 subjects (48 eyes) who had had a migraine within 2 weeks prior to testing. Individual %AUCs were then derived for several additional measures, specifically: the single worst performing (relative to normal) region in each visual field, the means of the worst 2 regions, the worst 3, the worst 4, etc. This allowed assessment of diagnostic performance as a function of the number of regions considered. We also derived ROCs for individual regional values for each eye as well as the difference between the mirror-image regions of the two eyes (generating 44 local ‘asymmetry’ measures/subject). For these ROCs, the largest %AUC was for the single worst region of each field in the ‘asymmetry’ assessment of YP (77.5% ± 5.13%). AUC values for YP asymmetry also remained relatively high for the means of the worst 5 regions in each field (76.7% ± 4.93). This indicated that YP fields contained a larger number of diagnostically meaningful regions than BP fields. For subjects whose migraine had occurred within 14 days earlier the %AUCs ranged from 52.8 to 65.4%.

**Table 6:** %AUCs ± SE for blue and yellow protocols comparing the 24 normal controls and the 24 patients experiencing migraine within 14 or fewer days before mfPOP testing

	Blue protocol (%)	Yellow protocol (%)
**AmpStd**		
**. Worst region of 44**	63.6 ± 5.97	66.6 ± 5.65
**. Mean of worst 5 regions**	59.8 ± 6.09	65.2 ± 5.74
**Asymmetry between eyes**		
**. Worst region of 44**	76.8 ± 5.05	77.5 ± 5.13
**. Mean of worst 5 regions**	70.5 ± 5.42	76.7 ± 4.93

## Discussion

This study’s three aims were to determine (i), whether BP was more likely than YP to exacerbate migraine, (ii) to determine whether there were any abnormalities of pupillary function following an attack of migraine, and (iii) to determine the power of mfPOP to distinguish patients with migraine from normal controls.

In relation to the first aim, there was no detectable increase in the incidence or severity of migraine headaches when using the blue stimulus, which had been specifically designed to stimulate the melanopsin-containing ipRGCs [14]. Regarding the second aim, at a global level the average sensitivity and times-to-peak of migraine patients did not differ from those of controls (Table 4). This finding is consistent with that of Cambron et al. [17], who examined patients during both ictal and interictal phases using standard pupillometry. However, looking at individual regions, we found significant reductions in regional sensitivities associated with a migraine attack occurring within 2 weeks before testing (Tables 5, 6 and Fig. 4). Regarding the third aim, the diagnostic power of those regional changes was modest but, at least for YP there were 5 or more regions of asymmetry between eyes per field that were quite diagnostic (Table 6) and about 5 inferotemporal regions of the YP fields showed consistent defects (Fig. 4).

Triptan use was associated with increases in per-region sensitivities. Changes in per-region times-to-peak were less consistent but there was possibly an increase in speed of response associated with a migraine attack occurring within the 2 weeks before testing. Given that time-to-peak was somewhat affected suggests that the triptans could have had an effect on cortical hyperexcitability [41] but this clearly requires further investigation.

As pointed out by [35, 36] at the background and stimulus used here pupil responses to blue stimuli are substantially driven by melanopsin, event to stimuli as short as 1 to 2 s. The times-to-peak for the BP were 100 ms longer than for YP (Table 4). This is in line with some melanopsin responses. We cannot rule out some rod intrusion however [35, 36]. The human melanopsin-driven response of the pupils can persist to over 2 Hz suggesting minimum latencies around 500 ms [42]. Responses of ipRGCs in dopaminergic amacrine cells can occur within 200 ms of light onset [7], much slower than rods or cones. The different types of ipRGCs differ in their physiology with M1 cells having the lowest thresholds and the fastest peak latencies at about 1 s [8].

It has recently been suggested that ipRGCs may be hyperactive in migraine [43], at least in persons with photophobia [5]. This might cause smaller pupils in such patients. Note that smaller pupils do not affect the relative measure AmpStd used here. The lack of any difference in pupil diameter between migraineurs and controls (Table 4) would indicate this was not happening here. That may be because a minority of our patients (15/38) reported any photosensitivity and no photophobia post-test. Absolute pupil diameter was significantly smaller for the blue stimuli (Table 4, t = 8.6, *p* < 0.0001), suggesting that the blue stimuli were driving melanopsin. It is possible however that the dynamic driving of ipRGC was not as great as other studies where photophobia was enhanced by strongly activating melanopsin [5, 43].

### Distribution of sensitivity and retinal changes

Abnormalities on flicker perimetry have been reported to persist for 7 days following an episode of migraine [19]. Of note, two of the migraine-induced flicker-fields in the literature demonstrated an inferotemporal defect [19, 20], similar to our findings (Fig. 4). These local visual field changes are unlikely to be due to generalized autonomic dysfunction of the pupil as this would be most likely to affect all regions of the visual field. Interestingly, the retinal nerve fibre layer (RNFL) of migraineurs can show thinning: a meta-analysis of six studies indicated that the thinning was most obvious superiorly, nasal retina being marginally more affected than temporal retina [44]. Superonasal retinal defects would correspond to the inferotemporal visual field defects found in this study. However, while an explanation at retinal level is possible, there may also be an effect at the cerebral cortex: pattern VEPs have been found to be abnormal in migraine [45, 46] while simultaneously-recorded pattern ERGs have not [45]. Interestingly, interictally recorded VEPs indicated hypersensitivity [45]. This would appear to agree with the recent work on ipRGCs and pupil responses during the interictal period [5]. Here we showed decreased sensitivity within 1 to 2 weeks after migraine attack for yellow stimuli, and for up to 60 days for blue (Table 5). Thus, migraine may represent an abnormally large fluctuation of ipRGC sensitivity pre- and post-migraine.

Perimetric defects reported for migraine tend to be sporadically distributed (like the BP fields here which averaged to zero), meaning that the Pattern Standard Deviation is often a better indicator of abnormality than the overall Mean Defect [19, 21, 22]. A similar effect has been observed in perimetric studies of concussion [47]. It is possible that both retinal and cortical effects contribute to the observed abnormalities, with YP being more sensitive to retinal abnormalities than BP [14]. Consistent with this is the observation that, in migraine, sensitivity to peripherally-presented contrast is more affected than contrast generated centrally [48]. A related finding is that alteration of peripheral contrast sensitivity has been shown to precede migraine onset by 1 to 2 days [49] though the stimuli of that study could not provide any information relating to any dominant contribution by one or more quadrants.

### mfPOP stimuli and visual distress

With the emergence of new modalities to treat migraine such as pharmacological manipulation of melanopsin [50] or blocking of blue wavelengths using tinted lenses [51], it is important to understand the effects of ipRGC stimulation on migraine pathophysiology. This study is the first to use a stimulus specifically designed to target the melanopsin-containing ipRGCs in order to look for an effect on migraine occurrence [14]. We compared this stimulus (BP) with an established stimulus designed to stimulate transient luminance pathways (YP). It is worth noting, however, that the BP stimulus design tended to reduce characteristics that could potentially cause discomfort in migraine subjects. For example, the stimuli were delivered randomly to different locations in the visual field rather than synchronously across the whole field, so each stimulus activated much less than 10% of the visual cortex [52]. Similarly, the stimuli did not contain stripes or checks [53], had smooth edges like sine-wave gratings, and contained no spatial frequencies above 2.0 cycles/deg. Thus, blue color and per-presentation stimulus-duration (1000 ms) were the only major characteristics that could have contributed to any effect that the BP stimuli might have had on triggering a migraine attack. We have used mfPOP stimuli that employed larger rectangular patterned stimuli [31], if these were blue and presented for longer durations it is possible that they could be a more effective probe of the contribution of ipRGCs to migraine.

## Conclusions

This study has demonstrated that stimulation of melanospin-containing ipRGCs did not alter migraine parameters. Localized field defects were detected if testing was carried out within 2 weeks after an attack. It also suggested that mfPOP may prove to be an important tool to study visual pathophysiology in migraineurs in the future.

## Data Availability

The datasets used and/or analysed during the current study available from the corresponding author on reasonable request.

## Abbreviations

AUC: Area under the curve
CSD: Cortical spreading depression
ipRGCs: Intrinsically-photosensitive retinal gangion cells
mfPOP: Mulifocal pupillographic objective perimetry
mfVEP: Multifocal visual evoked potential
OFA: ObjectiveFIELD analyser
OTC: Over-the-counter medications
ROC: Receiver operating characteristic
RNFL: Retinal nerve fibre layer
